# Selective optogenetic modulation of the PBN terminals in the lateral hypothalamic area and basal forebrain regulates emergence from isoflurane anesthesia in mice

**DOI:** 10.1186/s12871-023-02294-8

**Published:** 2023-10-02

**Authors:** Kai Lu, Zhenhuan Wang, Ning Bai, Ziyu Zhao, Xinrong Zhao, Yun He

**Affiliations:** 1 Department of Anesthesiology, Shaanxi Provincial People’s Hospital, Shaanxi, China; 2 Shaanxi Provincial Key Laboratory of Infection and Immunity, Shannxi, China; 3 Laboratory of Neurobiology, School of Biomedical Engineering, Tianjin Medical University, Tianjin, China; 4 Department of Anesthesiology, Shannxi Provincial Cancer Hospital, Yanta District, 309 Yanta W Rd, Xi’An, 710063 Shaanxi China

**Keywords:** PBN-LH, PBN-BF, Optogenetics, Isoflurane, Mechanism of anesthesia

## Abstract

While the mechanism of general anesthesia has been extensively studied, the underlying neural circuitry has yet to be fully understood. The parabrachial nucleus (PBN) plays a crucial role in modulating wakefulness and promoting arousal from general anesthesia. However, the specific role of PBN projections in the process of general anesthesia remains unclear. In this study, we bilaterally injected AAV-associated viruses encoding excitatory or inhibitory optogenetic probes into the PBN and implanted optical fibers in the LH or BF area. After four weeks, we optogenetically activated or inhibited the PBN-LH and PBN-BF pathways under 1.5 vol% isoflurane. We calculated the time it took for anesthesia induction and emergence, simultaneously monitoring changes in the burst-suppression ratio using electroencephalogram recording. Our findings indicate that optogenetic activation of the PBN-LH and PBN-BF projections plays a significant role in promoting both cortical and behavioral emergence from isoflurane inhalation, without significantly affecting the induction time. Conversely, photoinhibition of these pathways prolonged the recovery time, with no notable difference observed during the induction phase.

In summary, our results demonstrate that the PBN-LH and PBN-BF pathways are crucial for promoting arousal from isoflurane general anesthesia, but do not have a pronounced impact on the induction phase.

## Introduction

Despite its nearly two centuries of clinical use, the neural circuit mechanisms responsible for the effects of commonly used anesthetics in operating rooms remain incompletely understood. Recent research indicates that neural nuclei and circuits involved in regulating the sleep–wake cycle may have a role to play in the alteration of consciousness that occurs during general anesthesia [1, 2]. Hence, contemporary research on neural circuit mechanisms in general anesthesia primarily centers on brain regions responsible for controlling sleep and wakefulness. For example, various neurotransmitters, such as glutamatergic [3], dopaminergic [1], cholinergic [4], orexinergic [5], histaminergic [6] and noradrenergic [7] arousal projections, have been demonstrated to be involved in the induction or emergence of general anesthesia. Nevertheless, many nuclei or circuits in the brain that contribute to general anesthesia remain to be elucidated.

The parabrachial nucleus (PBN) is situated in the dorsolateral pons and houses numerous glutamatergic neurons that project extensively to various regions in the brain associated with promoting wakefulness. These regions encompass the lateral hypothalamus (LH), thalamus, basal forebrain (BF), amygdala complex, and cerebral cortex [8, 9]. Consequently, the PBN plays a pivotal role in initiating and sustaining EEG arousal and behavioral wakefulness [10]. Chemogenetic stimulation of the PBN, as well as the PBN-LH and PBN-BF pathways, is known to enhance wakefulness, while lesions of the PBN can result in significant EEG and behavioral unresponsiveness [8, 11]. Recent research has also demonstrated that electrical or chemogenetic stimulation of the PBN nucleus can lead to reawakening from isoflurane or propofol-induced general anesthesia [12, 13]. However, it's important to note that not all parts of the PBN are involved in regulating sleep or general anesthesia. It has been established that the medial PBN has a more significant impact on wakefulness compared to the lateral PBN [14]. Conversely, lesions in the medial PBN tend to induce more sleep than lesions in the lateral PBN [10]. These findings indicate that the medial PBN plays a crucial role in modulating both the sleep–wake system and the anesthesia-arousal cycle. Nevertheless, the precise role of medial PBN projections to other brain regions in general anesthesia remains to be fully elucidated.

The lateral hypothalamus (LH) and basal forebrain (BF) are two widely recognized pivotal nodes in the arousal system, receiving dense projections from the PBN [15, 16]. Numerous prior studies have demonstrated that these two nuclei play vital roles in the state of general anesthesia induced by various anesthetic agents [17–20]. Moreover, the chemogenetic stimulation of the PBN-LH and PBN-BF pathways elicits robust cortical arousal and promotes behavioral wakefulness [8]. Additionally, activation of the PBN leads to a noticeable increase in c-fos expression in the BF and LH during general anesthesia [13].

Previous evidence has predominantly depended on pharmacological or fluorescent staining techniques, which pose challenges in elucidating the interactions between PBN neurons and other brain regions during general anesthesia in live organisms. To address this issue, we leverage the advantage of millisecond precise in vivo control of optogenetic technology to explore the role of medial PBN-LH and PBN-BF pathways during in isoflurane anesthesia by exciting and inhibiting PBN axon terminals in the LH and BF.

## Materials and methods

### Animals

Male C57BL/6 J mice (8–10 weeks, 20∼25 g) were applied in this study. The animals were allowed to access water and food freely and were housed under the condition of light reverse cycle (lights on from 7:00 p.m. to 7:00 a.m). The room temperature and humidity were 23◦C ± 2◦C and 50% ± 10%, respectively. All the experimental processes were conducted during the light period and were performed in compliance with National Institutes of Health guidelines and overseen by the Animal Care and Use Committee of Tianjin Medical University.

### Surgical procedures

The mice were securely positioned in a stereotaxic frame (RWD, Shenzhen, China) and administered 1.5% isoflurane mixed with oxygen (at a flow rate of 1.0 L/min) to minimize any discomfort. Ophthalmic ointment was applied to their eyes to prevent dryness, while a heating blanket was employed to maintain a constant body temperature. Subsequently, the skin and fascia were gently removed from the skull, and the alignment of the bregma and lambda points was carefully ensured. Small craniotomies, with a diameter of 300–400 µm, were created bilaterally above the sites of virus injection and fiber implantation. Next, adeno-associated viruses (150nL) expressing channelrhodopsin 2 (rAAV2/9-CaMKIIa-hChR2(H134R)-EYFP-WPRE-hGH polyA, PT-0296), eNpHR 3.0 (rAAV2/9-CaMKIIa-eNpHR3.0-EYFP-WPRE-hGH polyA, PT-0008) or control virus EYFP (rAAV2/9-CaMKIIa-EYFP-WPRE-hGH polyA, PT-0012) were delivered into the PBN (anterior–posterior [AP]: -5.20 mm, medial–lateral [ML]: ± 1.20 mm, and dorsal–ventral [DV]: -2.5 mm) through a glass micropipette using a micro syringe pump at 30nL/min. Following the injection, the micropipette was kept stationary for about 10 min to ensure the virus was diffused sufficiently. Then three stainless-steel screws were anchored to the mouse skull as electroencephalogram (EEG) electrodes: AP –1.5 mm, ML + 1.5 mm, AP + 1.5 mm, ML –1.5 mm, and AP –5.5 mm, ML 0 mm. For optogenetic testing, optic fibers (200um in diameter) threaded through a ceramic ferrule were implanted bilaterally over the BF (AP: + 0.1 mm, ML: ± 1.3 mm, DV: -5.4 mm) or LH (AP: -0.94 mm, ML: ± 1.2 mm, DV: -4.4 mm) sites. Finally, dental cement was used to secure the skull's optic fibers and EEG electrodes. Following the surgery, the mice were given an intraperitoneal injection of meloxicam (0.03 mg/kg) for three consecutive days to minimize pain and discomfort. Each mouse was housed separately and allowed to recover for four weeks before undergoing assessments for the righting reflex and EEG activity.

### Examination of induction and emergence times

The mouse was placed within a transparent rectangular glass enclosure measuring 20 × 10 × 10 cm^3^, which was connected to an isoflurane vaporizer. The vaporizer's outflow was linked to an anesthetic gas recovery system. The mouse was granted a 10-min period to freely explore the interior of the box, enabling it to acclimate to the presence of electrode wires, fibers, and the experimental environment. Subsequently, the mouse was anesthetized using 1.5% isoflurane in oxygen (at a flow rate of 1 L/min). Once the anesthesia took effect, we rotated the glass box by 90 degrees every 15 s until the mouse was no longer able to turn over onto all four limbs, indicating a loss of the righting reflex (LORR). The administration of isoflurane was halted after maintaining anesthesia for 30 min. Subsequently, the glass box was rotated by 90 degrees every 15 s until the mouse regained its natural body posture, signifying the recovery of the righting reflex (RORR). It is widely accepted that the times for LORR and RORR serve as standard measures for assessing the initiation and resolution of general anesthesia in mice. Throughout the period of general anesthesia maintenance, the mouse's body temperature was carefully maintained using a heating blanket.

### Optogenetics

Optogenetic tests were conducted at least four weeks after the injection of the optogenetic virus. A ceramic ferrule was connected to a laser diode (Thinker Tech Nanjing Biotech Co., Ltd, China) via a fiber. To facilitate optogenetic stimulation, a 473-nm blue-light laser was used, and the light intensity at the tip of the optical fiber cannula was adjusted to deliver 20-30mW/mm^2^ using a power calibrator (SANWA Electric Instrument, Tokyo, Japan) before the experiment. Programmed light pulse trains (20 ms in duration, 30 Hz frequency, and 10 s-ON/10 s-OFF cycle) were utilized to stimulate the PBN-LH or PBN-BF pathways. Conversely, to silence the PBN-LH or PBN-BF pathways, 593-nm yellow-light laser pulse were continuously delivered for optogenetic inhibition [21]. Throughout the induction phase, the mice received optical stimulation from the moment they began inhaling isoflurane until they reached LORR. Light pulse trains were also administered during the emergence phase, as the isoflurane anesthesia was being discontinued, until RORR was attained. Furthermore, behavioral and EEG changes were continuously monitored during the optogenetic experiments.

### EEG recording and analysis

The EEG signals were recorded via three stainless screws fixed on the mouse skull by the PowerLab 16/35 amplifier system (PL3516, AD Instruments, Bella Vista, NSW, Australia) and LabChart Pro V8.1.13 software (MLU60/8, AD Instruments). The raw EEG data were digitized at 1000 Hz and bandpass filtered at 0.3–50 Hz for analysis. MATLAB (R2014a; MathWorks, Natick, MA, USA) was used to conduct burst-suppression ratio (BSR) and spectrum drawing. The EEG threshold was set based on the amplitude of the each mouse's suppression waves (basically less than 10 µV), and the suppression wave's minimum duration was no less than 0.5 s [22]. If the amplitude of EEG was lower than the threshold, and the amplitude was a suppressed event assigned a value of 1. Otherwise, the amplitude of EEG was higher than the threshold, the amplitude was a burst event with a value of 0. Finally, the BSR was calculated based on the percentage of suppression events for 2 min before and during optical stimulation.

### Histological verification and immunohistochemistry

After achieving deep anesthesia, mice underwent transcardial perfusion with 4% paraformaldehyde (PFA) in phosphate-buffered saline (PBS) to confirm viral expression and the positioning of fiber implants, as well as c-fos expression. The brain samples were left in 4% PFA at 4 °C overnight, followed by soaking in 30% sucrose in PBS at 4 °C until they sank. Subsequently, the brains were coronally sectioned into 30 μm slices using a freezing microtome (LEICA, CM1950).

For c-fos staining experiments, mice brains were collected 90 min after the completion of photoactivation. Brain sections containing LH and BF were initially immersed in a PBS solution (pH 7.4) containing 5.0% normal goat serum and 0.3% TritonTM X-100 for two hours at room temperature. They were then incubated overnight at 4 °C with a rabbit c-Fos antibody (CST, 1:1000), followed by three 10-min washes with PBST (PBS with 0.1% Triton X-100, vol/vol). Subsequently, the sections were incubated with a diluted secondary antibody (goat anti-rabbit conjugated to Alexa 568, 1:500 dilution) at room temperature for 1 h in the dark. The slides were then washed twice with PBST for 5 min and incubated with DAPI (5 mg/ml, 1:1000) for 5 min. DAPI was washed off three times with PBST for 2 min. Images were captured using a fluorescence microscope (BX51, Olympus).

### Statistical analysis

The EEG and cfos histology data were collected and analyzed in this experiment using a blind method. The statistical analysis were performed by Prism 9.0 (GraphPad Software Inc., CA, USA). The results of c-fos density and BSR differences in EEG spectral power were compared with Student’s t-test, and two-way ANOVA followed by Bonferroni correction was applied to analyze the optogenetic behavioral tests.

All data are represented as the mean ± the standard deviation of the mean (SD), and *P* < 0.05 was considered statistically significant.

## Results

### Optical stimulation of PBN-LH pathway facilitates emergence from isoflurane anesthesia

**Fig. 1:**
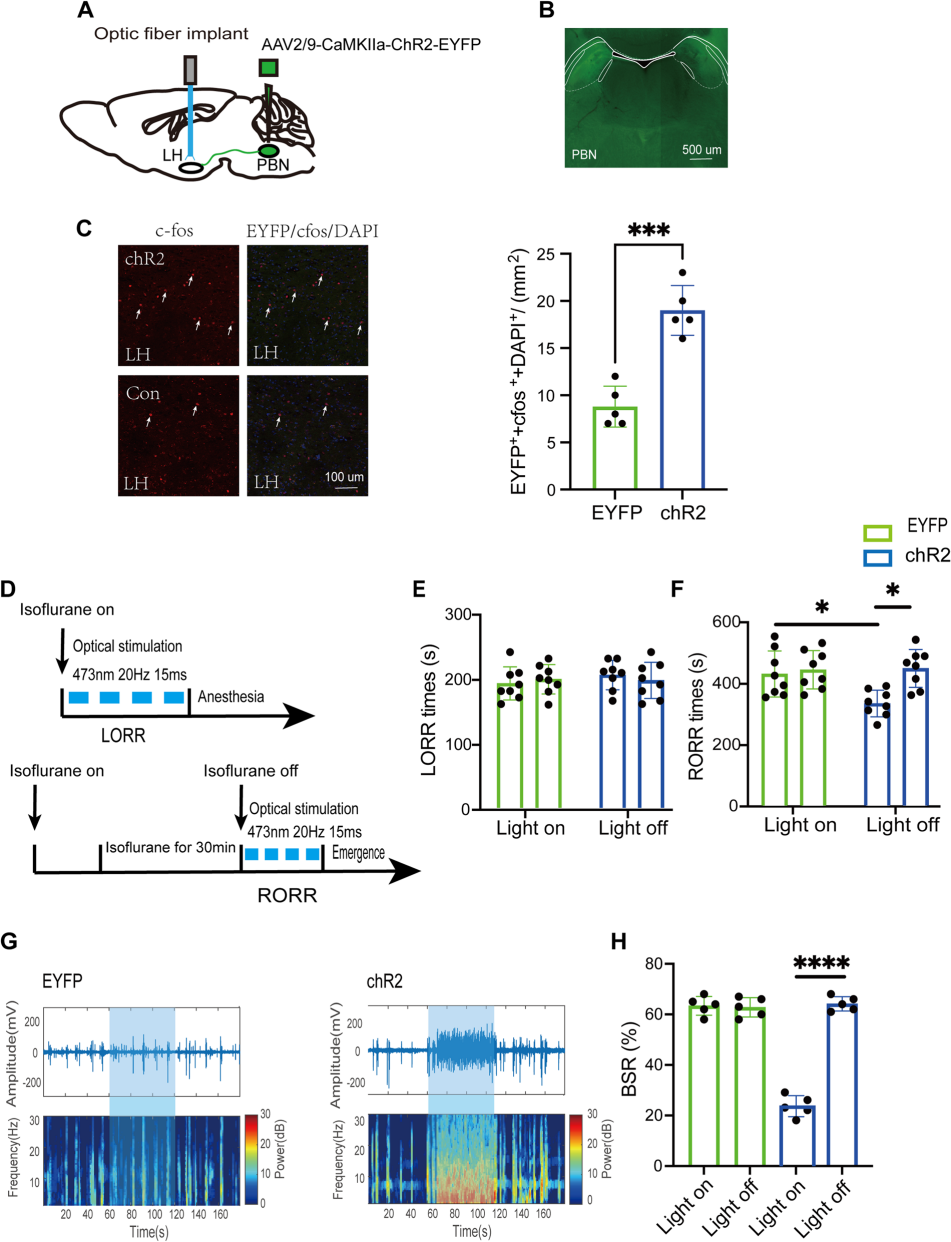
Optical stimulation of PBN-LH projections accelerates emergence from isoflurane anesthesia. **A** Schematic of excitatory optogenetic virus (ChR2) injection into the PBN and optical implant into the LH in mice. **B** Histological and fluorescence image showing the virus expression in the PBN. **C** Representative images (left) and quantification (right) of EYEP (green), c-Fos (red), and DAPI (blue) colabeled in the LH, n = 5 per group. **D** Schematic showing the protocol of optogenetic activation of PBN-LH projections during isoflurane anesthesia. **E** and **F** LORR time and RORR time of mice with optogenetic activation of PBN-LH projections, *n* = 8 per group. **G** Representative EEG traces (above) and corresponding power spectra (below) optical activation under 1.5% isoflurane anesthesia in the EYEP (left) and ChR2 group (right). **H** Statistics of the change of BSR before and during optical stimulation, *n* = 5 per group. **P < 0.01, ***P < 0.001, ****P < 0.0001, data are graphed as mean ± SD

Following the bilateral injection of the optogenetic virus (ChR2) into the PBN and the implantation of optical fibers into the LH for the activation of the PBN-LH pathway (Fig. 1A), virus expression was assessed post-experimentally (Fig. 1B). The neural activity marker c-fos was also employed to confirm the effectiveness of LH neuron activation. The findings revealed that optical stimulation notably elevated c-fos densities in LH neurons within the ChR2 group when compared to the control EYFP group (ChR2: 19.0 [2.6] vs. EYFP: 8.8 [2.2], *p* = 0.0002, n = 5, Fig. 1C).Continuous stimulation was applied throughout the induction and emergence phases of isoflurane anesthesia (Fig. 1D). In comparison to the control EYFP group, optogenetic activation of the PBN-LH pathway notably decreased the emergence time. (ChR2-light-on: 314.4 [41.0] s vs. EYFP-light-on: 405.4 [71.1] s, *p* = 0.0342, *n* = 8, Fig. 1F), while there was no notable difference in the induction time (ChR2-light-on: 203.5 [22.7] s vs. EYFP-light-on: 191.6 [25.4] s, *p* = 0.6561, *n* = 8, Fig. 1E). Within the ChR2 group, photostimulation also shortened the RORR time (ChR2-light-on: 314.4 [41.0] s vs. ChR2-light-off: 422.5 [58.5] s, *p* = 0.0229, *n* = 8, Fig. 1F) and did not affect the LORR time (ChR2-light-on: 203.5 [22.7] s vs. ChR2-light-off: 196.3 [27.9] s, *p* = 0.596, *n* = 8, Fig. 1E) compared to the ChR2-light-off control. Furthermore, compared with the control EYFP group, the EEG recordings demonstrated activation of the PBN-LH projection dramatically reduced the burst suppression pattern (Fig. 1G). The BSR in the ChR2 group obviously decreased from 64.2% ± 2.8% to 20.6% ± 4.2% (*P* < 0.0001, *n* = 5, Fig. 1H).

### Optical inhibition of PBN-LH pathway delays emergence from isoflurane anesthesia

**Fig. 2:**
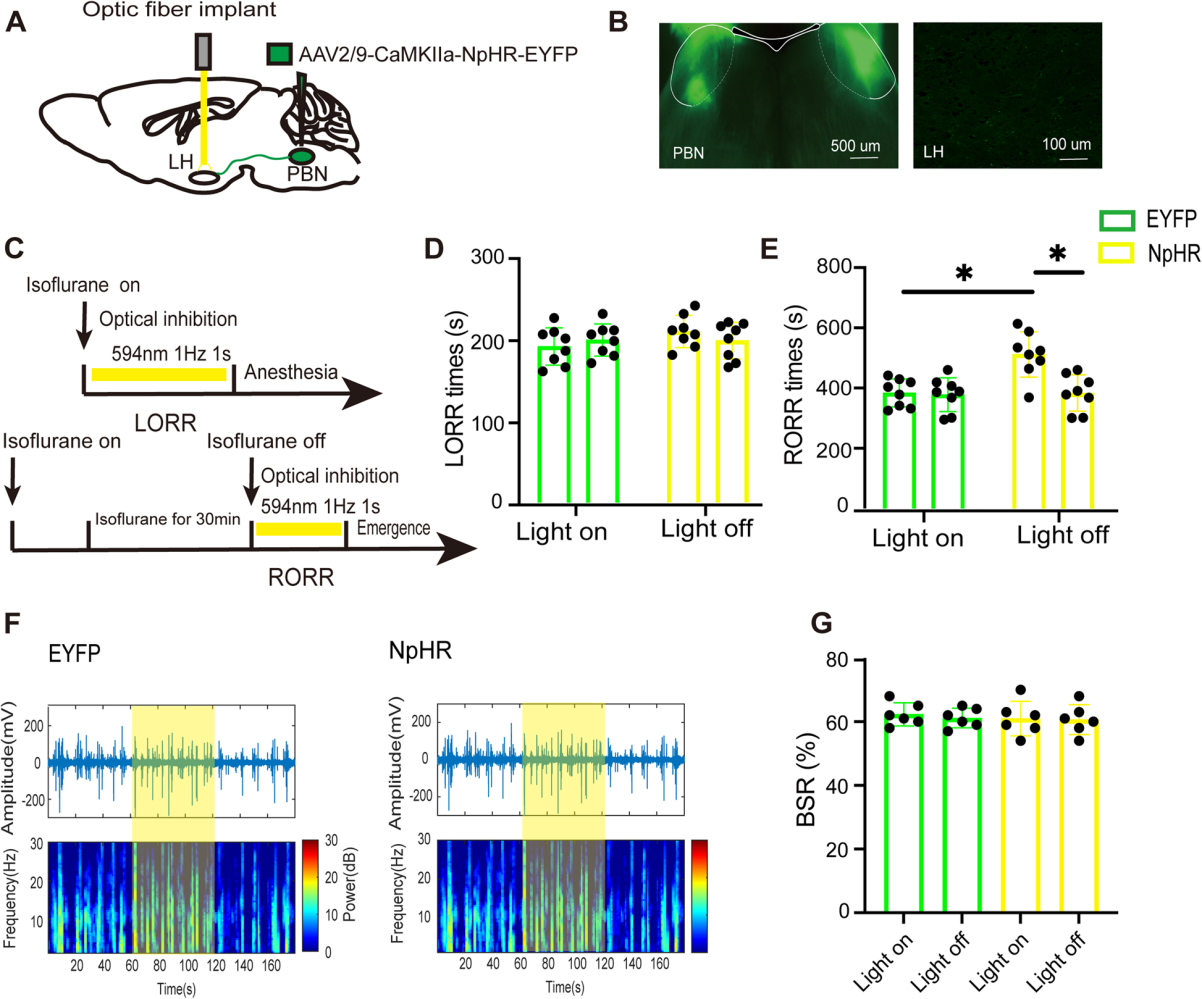
Optical inhibition of PBN-LH projections prolongs emergence from isoflurane anesthesia. **A** Schematic of inhibitory optogenetic virus (NpHR) injection into the PBN and optical implant into the LH in mice. **B** Histological and fluorescence image showing the virus expression in the PBN and LH. **C** Schematic showing the protocol of optogenetic suppression of PBN-LH projections during isoflurane anesthesia. **D** and** E** LORR time and RORR time of mice with optogenetic inhibition of PBN-LH projections, n = 8 per group. **F** Representative EEG traces (above) and corresponding power spectra (below) optical activation under 1.5% isoflurane anesthesia in the EYEP (left) and NpHR group (right). **G** Statistics of the change of BSR before and during optical suppression, *n* = 6 per group. **P < 0.01, data are graphed as mean ± SD

The optogenetic virus (NpHR) was bilaterally injected into the PBN and followed by implanting optic fibers into the LH of the mouse (Fig. 2A). After 4 weeks, the inhibitory photosensitive protein was expressed in the LH (Fig. 2B). Yellow laser light was administrated during both the induction and emergence periods of isoflurane anesthesia (Fig. 2C). Compared with the control EYFP group, optogenetic inhibition of PBN-LH projection showed almost no difference in the LORR time (NpHR-light-on: 207.9 [19.9] s vs. EYFP-light-on: 189.1 [14.9] s, *p* = 0.1005, *n* = 8, Fig. 2D). However, it significantly prolonged the emergence time (NpHR-light-on: 500.8 [73.8] s vs. EYFP-light-on: 381.8 [47.6] s, *p* = 0.0448, *n* = 8, Fig. 2E). Within the NpHR group, compared with the NpHR-light-off control, photoinhibition did also not affect the LORR time (NpHR-light-on: 207.9 [19.9] s vs. NpHR-light-off: 193.8 [25.5] s, *p* = 0.4607, *n* = 8, Fig. 2D) but prolonged the RORR time (NpHR-light-on: 500.8 [73.8] s vs. ChR2-light-off: 375.1 [59.0] s, *p* = 0.041, *n* = 8, Fig. 2E). What’s more, the EEG recordings suggested suppression of the PBN-LH pathway had no influence in the BSR (NpHR-light-on: 61 [5.4] s vs. NpHR-light-off: 60.6 [4.7] s, *p* = 0.9087, *n* = 5, Fig. 2F, G).

### Optical stimulation of PBN-BF pathway facilitates emergence from isoflurane anesthesia

**Fig. 3:**
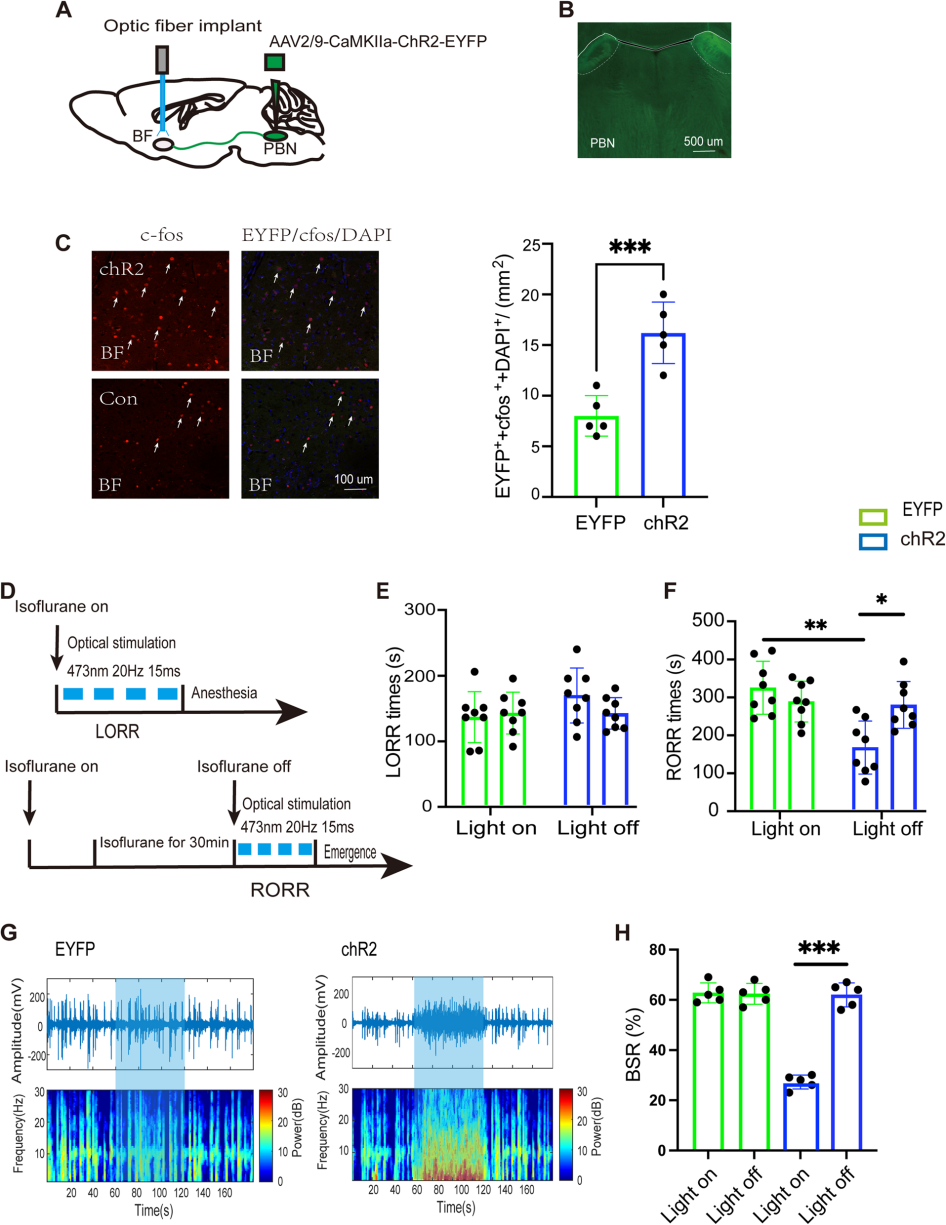
Optical stimulation of PBN-BF projections accelerates emergence from isoflurane anesthesia. **A** Schematic of excitatory optogenetic virus (ChR2) injection into the PBN and optical implant into the BF in mice. **B** Histological and fluorescence image showing the virus expression in the PBN. **C** Representative images (left) and quantification (right) of EYEP (green), c-Fos (red), and DAPI (blue) colabeled in the BF, *n* = 5 per group. **D** Schematic showing the protocol of optogenetic activation of PBN-BF projections during isoflurane anesthesia. **E** and **F** LORR time and RORR time of mice with optogenetic activation of PBN-BF projections, n = 8 per group. **G** Representative EEG traces (above) and corresponding power spectra (below) optical activation under 1.5% isoflurane anesthesia in the EYEP (left) and ChR2 group (right). **H** Statistics of the change of BSR before and during optical stimulation, *n* = 5 per group. **P < 0.01, ***P < 0.001, data are graphed as mean ± SD

In our pursuit of a deeper understanding of the PBN's role in regulating consciousness levels during general anesthesia, we also manipulated the activity of the projection from the PBN to the BF using optogenetics. To explore this hypothesis, we injected the optogenetic virus (ChR2) into the PBN and implanted optical fibers into the BF to activate the PBN-BF pathway (Fig. 3A). The expression of the virus was assessed following the experiments (Fig. 3B), and c-fos staining was conducted to validate the heightened neural activity in BF neurons in response to optical stimulation within the ChR2 group (ChR2: 16.2 [3.0] vs. EYFP: 8.0 [2.0], *p* = 0.001, *n* = 5, Fig. 3C). As previously, stimulation was delivered continuously during the induction and emergence periods of isoflurane anesthesia (Fig. 3D). Compared with the control EYFP group, optogenetic activation of PBN-BF notably shortened the RORR time (ChR2-light-on: 341.0 [32.4] s vs. EYFP-light-on: 390.6 [41.1] s, *p* = 0.0339, *n* = 8, Fig. 3F), while there was no obvious difference in the LORR time (ChR2-light-on: 212.5 [28.3] s vs. EYFP-light-on: 190.4 [26.2] s, *p* = 0.727, *n* = 8, Fig. 3E). Within the ChR2 group, photostimulation decreased the emergence time (ChR2-light-on: 329.6 [34.1] s vs. ChR2-light-off: 384.4 [30.2] s, *p* = 0.0214, *n* = 8, Fig. 3F) and did not affect the induction time (ChR2-light-on: 212.5 [28.3] s vs. ChR2-light-off: 194.1 [16.2] s, *p* = 0.9218, *n* = 8, Fig. 3E) compared to the ChR2-light-off control. Subsequently, in comparison to the control EYFP group, EEG recordings revealed that activation of the PBN-BF projection significantly reduced the burst suppression pattern (Fig. 3G). The Burst Suppression Ratio (BSR) in the ChR2 group notably decreased from 62.0% ± 4.7% to 22.8% ± 3.5% (*P* = 0.0002, *n* = 5, Fig. 3H).

### Optical inhibition of PBN-BF pathway delays emergence from isoflurane anesthesia

**Fig. 4:**
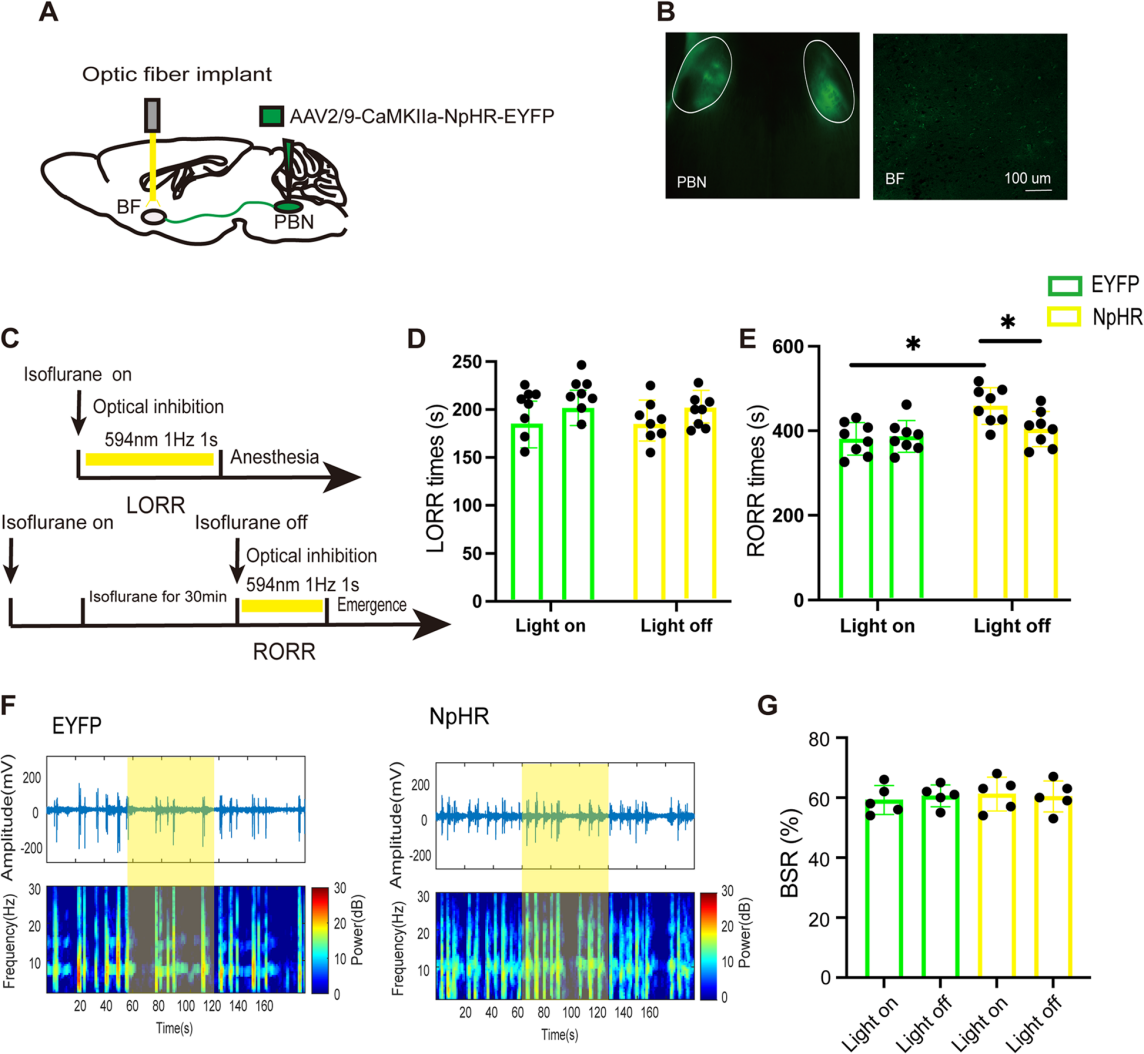
Optical inhibition of PBN-BF projections delays emergence from isoflurane anesthesia. **A** Schematic of inhibitory optogenetic virus (NpHR) injection into the PBN and optical implant into the BF in mice. **B** Histological and fluorescence image showing the virus expression in the PBN and BF. **C** Schematic showing the protocol of optogenetic suppression of PBN-BF projections during isoflurane anesthesia. **D** and** E** LORR time and RORR time of mice with optogenetic inhibition of PBN-BF projections, *n* = 8 per group. **F** Representative EEG traces (above) and corresponding power spectra (below) optical activation under 1.5% isoflurane anesthesia in the EYEP (left) and NpHR group (right). **G** Statistics of the change of BSR before and during optical suppression, *n* = 6 per group. *P < 0.05, **P < 0.01, data are graphed as mean ± SD

To specifically inhibit the PBN-BF pathway, we bilaterally injected the optogenetic virus (eNpHR) into the PBN and implanted optic fibers into the BF of the mouse (Fig. 4A). The location of virus expression are shown in (Fig. 4B). Yellow laser light was delivered during both the induction and emergence periods of isoflurane anesthesia (Fig. 4C). Compared with the control EYFP group, optogenetic inhibition of PBN-BF projection showed almost no difference in the LORR time (NpHR-light-on: 182.8 [21.4] s vs. EYFP-light-on: 183.3 [24.3] s, *p* = 0.9658, *n* = 8, Fig. 4D). However, it significantly prolonged the emergence time (NpHR-light-on: 452.1 [43.4] s vs. EYFP-light-on: 387.3 [44.9] s, *p* = 0.0404, *n* = 8, Fig. 4E). Within the NpHR group, compared with the NpHR-light-off control, photoinhibition did not affect the LORR time (NpHR-light-on: 182.8 [21.4] s vs. NpHR-light-off: 198.6 [17.1] s, *p* = 0.9005, *n* = 8, Fig. 1D) and prolonged the RORR time (NpHR-light-on: 452.1 [43.4] s vs. NpHR-light-off: 397.6 [41.6] s, *p* = 0.0443, *n* = 8, Fig. 4E). What’s more, the EEG recordings suggested supression of the PBN-BF ppathway had no influence in the BSR (NpHR-light-on: 61.2 [5.6] s vs. NpHR-light-off: 60.4 [5.2] s, *p* = 0.7371, *n* = 5, Fig. 4F, G).

## Discussion

The PBN plays a crucial role in the regulation of sleep and emergence from general anesthesia. To investigate the specific effects of PBN neurons on the BF and LH during anesthesia emergence, we used optogenetic approaches to bidirectionally manipulate the PBN-LH and PBN-BF projections in isoflurane anesthesia. We found that targeted activation of these pathways did not significantly affect the induction time but resulted in differences in both cortical and behavioral performance during emergence. These results highlight the important role of the PBN projections in the emergence of isoflurane anesthesia.

Optogenetics is a cutting-edge technique that enables precise manipulation of specific neurons or pathways in living animals with high temporal resolution [23]. This method has been effectively integrated with behavioral paradigms in animal studies to investigate the roles of distinct neuron subtypes or their afferent/efferent circuits in various brain functions. For instance, it has been employed to examine cognition-related neurons in the locus coeruleus nucleu [24] and depression responsive medium spiny neurons in the NAc [25]. Given the swift transitions between states of anesthesia, we employed this technique to explore the downstream systems regulated by the PBN. Since the loss of consciousness is a fundamental aspect shared by both sleep and general anesthesia, it has been proposed that these two states have overlapping neural endpoints. Research has demonstrated that the reversible loss and recovery of consciousness induced by anesthetics result from the precise regulation of various arousal or sleep nuclei [26].

The PBN is known to play a crucial role in regulating arousal, as injury to the PBN region can result in coma or persistent vegetative state in both humans and animals [27, 28]. Studies have also demonstrated that specific activation of the PBN can interrupt sleep and promote wakefulness [8], and reliably accelerate emergence from isoflurane general anesthesia [12]. In addition, even chemogenetic activation of PBN astrocytes increased wakefulness amount and shortened emergence time from isoflurane anesthesia [29]. Furthermore, the PBN sends out abundant neural projections to innervate the arousal-promoting BF and LH regions [30], which have also been implicated in consciousness transition during general anesthesia [31]. Recently, both PBN-BF and PBN-LH pathways were verified to play essential role in controlling wakefulness which was reflected by arousal effect from sleep after activating the two neuronal circuits [14]. Consistent with the above findings, using optogentic technique in the current study, our results further demonstrated that specific stimulation of both the PBN-LH and PBN-BF pathways in mice accelerated the emergence of righting reflex and induced a pronounced decrease in burst-suppression ratio during isoflurane general anesthesia. However, we failed to observe significant changes in the induction time after activation of the two neural circuits.

On the contrary, specific photoinhibition of the PBN-LH and PBN-BF pathways prolonged the emergence time despite no obvious difference in burst-suppression ratio. Again, there was no significant changes in the induction time. These results are in line with previous studies that focusing on the role of PBN neurons in isoflurane general anesthesia. Luo et al. recorded the calcium signals in vivo in real-time and found that PBN neural activity is inhibited during anesthesia but is significantly activated during the recovery from propofol and isoflurane anesthesia. Using chemogenetic methods, they also discovered that PBN neuron activation reduced the recovery time but did not affect the induction time [13]. Together, these interesting findings suggest that the neural mechanisms underlying the transition into and out of the anesthetized state may not be identical. One possible explanation is that the induction and resuscitation of isoflurane anesthesia have specific neural targets. However, another possibility is about the experiment design, because we only start optogenetic modulation at the same time as isoflurane inhalation. It is probable that delayed induction happens when optogenetic stimulation performs earlier than isoflurane delivery. Additionally, of note, the effect of PBN during the induction phase of general anesthesia appears to be flexible under different anesthetics. For instance, Luo et al. reported that chemogenetic activation of PBN glutamatergic neurons not only significantly reduced the emergence time caused by sevoflurane anesthesia but also increased the induction time and the ED50 of sevoflurane [3]. Similar interesting observations have also been made in the study of LH orexinergic neurons during general anesthesia. A recent study suggested that despite the fact that LH orexinergic neurons could regulate maintenance and emergence from both isoflurane and desflurane inhalation, their role differs considerably in their actions during the induction phases of isoflurane versus desflurane [32]. This unique phenomenon indicates that different anesthetics may also target specific neural endpoints or pathways during the induction stage. This exciting discovery could further explore the mechanism of action of different anesthetics in the future and help solve relevant clinical problems.

The present study has several limitations that warrant consideration. Firstly, we did not observe a significant correlation between the modulation of the two PBN projections and the induction time of isoflurane. This lack of effect may be attributed to our experimental design, as we did not initiate activation prior to isoflurane inhalation, and we did not explore the effects with other types of anesthetics. Additionally, we did not investigate whether either the PBN-LH or PBN-BF pathway plays a more critical role in facilitating recovery from isoflurane anesthesia. Lastly, our study exclusively involved male mice, however, gender differences may potentially impact the results of this experiment. As a result, our future research will prioritize addressing these aforementioned limitations.

In summary, our findings support the hypothesis that the PBN projections play an essential role in regulating emergence from isoflurane anesthesia.

## Data Availability

The data that support the findings of the present study are available from the corresponding authors upon reasonable request.

## References

[1] Taylor NE (2016). Optogenetic activation of dopamine neurons in the ventral tegmental area induces reanimation from general anesthesia. Proc Natl Acad Sci U S A.

[2] Mashour GA, Pal D (2012). Interfaces of sleep and anesthesia. Anesthesiol Clin.

[3] Wang TX (2019). Activation of Parabrachial Nucleus Glutamatergic Neurons Accelerates Reanimation from Sevoflurane Anesthesia in Mice. Anesthesiology.

[4] Kenny JD (2016). Physostigmine and Methylphenidate Induce Distinct Arousal States During Isoflurane General Anesthesia in Rats. Anesth Analg.

[5] Wang D (2021). Selective optogenetic activation of orexinergic terminals in the basal forebrain and locus coeruleus promotes emergence from isoflurane anaesthesia in rats. Br J Anaesth.

[6] Luo T, Leung LS (2011). Involvement of tuberomamillary histaminergic neurons in isoflurane anesthesia. Anesthesiology.

[7] Vazey EM, Aston-Jones G (2014). Designer receptor manipulations reveal a role of the locus coeruleus noradrenergic system in isoflurane general anesthesia. Proc Natl Acad Sci U S A.

[8] Qiu MH (2016). Stimulation of the Pontine Parabrachial Nucleus Promotes Wakefulness via Extra-thalamic Forebrain Circuit Nodes. Curr Biol.

[9] Saper CB, Loewy AD (1980). Efferent connections of the parabrachial nucleus in the rat. Brain Res.

[10] Fuller PM (2011). Reassessment of the structural basis of the ascending arousal system. J Comp Neurol.

[11] Kaur S (2013). Glutamatergic signaling from the parabrachial nucleus plays a critical role in hypercapnic arousal. J Neurosci.

[12] Muindi F (2016). Electrical stimulation of the parabrachial nucleus induces reanimation from isoflurane general anesthesia. Behav Brain Res.

[13] Luo T (2018). Parabrachial Neurons Promote Behavior and Electroencephalographic Arousal From General Anesthesia. Front Mol Neurosci.

[14] Xu Q (2021). Medial Parabrachial Nucleus Is Essential in Controlling Wakefulness in Rats. Front Neurosci.

[15] Adamantidis AR (2007). Neural substrates of awakening probed with optogenetic control of hypocretin neurons. Nature.

[16] Xu M (2015). Basal forebrain circuit for sleep-wake control. Nat Neurosci.

[17] Zhang LN (2012). Orexin-A facilitates emergence from propofol anesthesia in the rat. Anesth Analg.

[18] Dong HL (2006). Orexins increase cortical acetylcholine release and electroencephalographic activation through orexin-1 receptor in the rat basal forebrain during isoflurane anesthesia. Anesthesiology.

[19] Zhang LN (2016). Orexin-A facilitates emergence of the rat from isoflurane anesthesia via mediation of the basal forebrain. Neuropeptides.

[20] Luo TY (2020). Basal Forebrain Cholinergic Activity Modulates Isoflurane and Propofol Anesthesia. Front Neurosci.

[21] Zhao S (2021). Lateral Hypothalamic Area Glutamatergic Neurons and Their Projections to the Lateral Habenula Modulate the Anesthetic Potency of Isoflurane in Mice. Neurosci Bull.

[22] Chemali J (2013). Burst suppression probability algorithms: state-space methods for tracking EEG burst suppression. J Neural Eng.

[23] Vlasov K, Van Dort CJ, Solt K (2018). Optogenetics and Chemogenetics. Methods Enzymol.

[24] Kempadoo KA (2016). Dopamine release from the locus coeruleus to the dorsal hippocampus promotes spatial learning and memory. Proc Natl Acad Sci U S A.

[25] Li Z (2018). Cell-Type-Specific Afferent Innervation of the Nucleus Accumbens Core and Shell. Front Neuroanat.

[26] Zhang K, Pan J, Yu Y (2022). Regulation of Neural Circuitry under General Anesthesia: New Methods and Findings. Biomolecules.

[27] Parvizi J, Damasio AR (2003). Neuroanatomical correlates of brainstem coma. Brain.

[28] Fischer DB (2016). A human brain network derived from coma-causing brainstem lesions. Neurology.

[29] Liu PC (2022). Parabrachial nucleus astrocytes regulate wakefulness and isoflurane anesthesia in mice. Front Pharmacol.

[30] Fulwiler CE, Saper CB (1984). Subnuclear organization of the efferent connections of the parabrachial nucleus in the rat. Brain Res.

[31] Laalou FZ (2008). Involvement of the basal cholinergic forebrain in the mediation of general (propofol) anesthesia. Anesthesiology.

[32] Zhao S (2021). Activation of Orexinergic Neurons Inhibits the Anesthetic Effect of Desflurane on Consciousness State via Paraventricular Thalamic Nucleus in Rats. Anesth Analg.

